# Perspectives on ageing acceptance among women in middle adulthood: a qualitative study

**DOI:** 10.1080/17482631.2025.2598720

**Published:** 2025-12-10

**Authors:** Goda Gegieckaitė, Gražina Rapolienė, Milda Kukulskienė, Jill Chonody, Barbra Teater

**Affiliations:** a The Department of Demographic and Family Research, Institute of Sociology, The Lithuanian Centre for Social Sciences, Vilnius, Lithuania; b The Department of Health Psychology, Lithuanian University of Health Sciences, Kaunas, Lithuania; c School of Social Work, College of Health Sciences, Boise State University, Boise, Idaho, USA; d Department of Social Work, College of Staten Island, City University of New York, New York City, New York, USA

**Keywords:** Middle adulthood, women, ageing acceptance, qualitative study, attitudes towards ageing

## Abstract

**Purpose:**

A dearth of qualitative studies explore what women in middle adulthood perceive as important influences on their attitudes toward ageing, particularly in Lithuania. A greater understanding of ageing acceptance can inform research and action to promote more positive ageing. This study of women in middle adulthood explored their perception of what helps or hinders ageing acceptance.

**Methods:**

Semi-structured interviews with 26 women aged 30–57 years were conducted. We adopted an inductive, experiential orientation within reflexive thematic analysis. Four main themes and 12 subthemes were developed.

**Results:**

“Examples of older figures in the environment” theme described older adults in participants' lives significantly contributing to the image of ageing. The theme “Personal attitudes and traits” discussed personal qualities that were related to ageing acceptance. The theme “(no) pressure in the environment” reflected others' perceptions of ageing influencing one's own ageing views. “Tension over control of ageing” theme illustrated the complicated nature of balancing control and acceptance in the ageing process.

**Conclusions:**

This study highlights the unique roles of the social environment, cultural pressures, internal conflicts, and experiences with older people in ageing acceptance. Interventions aimed at negative perceptions of ageing, better body and self-acceptance could help promote acceptance of ageing.

## Introduction

Ageing is developmental in nature and can be defined as the continuous process of growing older. The internal and external signs of ageing, signals from the environment that one is ageing or being perceived as older, may spark a range of reactions. It can cause significant negative emotional reactions for some, while others readily accept and embrace ageing (e.g., Chonody & Teater, 2016; Lasher & Faulkender, 1993). There is a lack of agreement on what acceptance exactly means (Mrazek et al., 2024; Williams & Lynn, 2010), but Wong et al. (1994), when describing death acceptance, named it entailing not only cognitive awareness but also a positive or at least neutral emotional reaction. While death acceptance is distinct from ageing acceptance, given their similarity as unavoidable existential issues, the same two components could be relevant in defining ageing acceptance. In this paper, we broadly conceptualize ageing acceptance as one's relatively positive or neutral attitudes toward ageing, including emotional and cognitive views and reactions to ageing. Consistently strong and negative views and reactions to ageing could show challenges in ageing acceptance. Acceptance is not equated to resigning or giving up, and research supports the idea of the paradox of acceptance of an experience—acceptance can lead to wanted change, and acceptance of frustrating experiences is associated with better mental well-being (Williams & Lynn, 2010). In terms of ageing, a similar pattern emerges in that more negative views and reactions to ageing are related to a decrease in health behaviours or ageing preparations (Korkmaz Aslan et al., 2017; Kornadt et al., 2015), worse mental health, physical health, and other quality of life outcomes (Westerhof et al., 2023; Wettstein et al., 2020). These connections are often explained by stereotype embodiment theory—people live and act according to their negative ageing stereotypes as they age (Levy, 2009). Given its impact on health and well-being, a better understanding of ageing perceptions could inform strategies that promote and encourage better attitudes towards and acceptance of ageing, which in turn could help people prepare, experience, and embrace different stages of life more positively.

In quantitative studies, people's perception and attitudes toward ageing have usually been explored not through acceptance but a number of related constructs with different emphases, such as ageing anxiety, attitudes towards ageing, self-perception of ageing, and so on (Kornadt et al., 2019; Wahl et al., 2022). Ageing anxiety is understood as a combined concern and anticipation of losses centred around the ageing process (Lasher & Faulkender, 1993), a construct emphasizing emotional reactions to ageing, while negative attitudes towards ageing can involve holding negative stereotypes about ageing in general, one's own ageing, or older adults (Bryant et al., 2016; Hess, 2006).

Understanding the ageing perceptions of adults in middle adulthood, the life stage between young adulthood and older age, is critical, as it is the time when people start to notice the first signs of their own ageing. Their attitudes towards ageing and how they cope with ageing anxiety are developing or solidifying, and this might set a course for their further ageing process. Previous studies have shown that ageing anxiety is higher among younger adults compared to their older counterparts (e.g., Barrett & Toothman, 2018), even though much of the ageing attitudes and ageing anxiety research has focused on older adults. Thus, understanding ageing perceptions in adults in middle adulthood is an important issue with potential implications for enhancing health and well-being. Furthermore, some evidence suggests that women in middle adulthood might experience ageing differently than men (Bodner et al., 2015; Brunton & Scott, 2015) as they might be more concerned about age-related changes in physical appearance, and menopause might introduce additional anxiety and awareness of their ageing (Brunton & Scott, 2015; Slevec & Tiggemann, 2010). Given the distinct societal pressures and concerns that women in middle adulthood face around their age-related changes, targeted research to understand factors related to their attitudes towards their ageing is needed.

Several types of factors framed within a few main theories are typically examined in quantitative studies as related to how a person views ageing and older age. One group of factors related to ageing anxiety is internalized negative societal stereotypes about older adults, ageism, and inaccurate knowledge about the ageing process (Cooney et al., 2021; Donizzetti, 2019). These connections are often explained within stereotype embodiment (Levy, 2009) and social identity theories (Tajfel & Turner, 1986)—people internalize a negative image of ageing and old age and do not want to belong to a socially stigmatized and less valued age group. Relatedly, the fear of a decrease in social value because of a changing appearance might also explain previous research findings that women's greater investment in appearance and the importance they place on appearance were associated with higher ageing anxiety (Carrard et al., 2021; Slevec & Tiggemann, 2010). More and better-quality contact with older people was found to be related to more positive attitudes toward ageing (Barnett & Adams, 2018; Bousfield & Hutchison, 2010), as it is thought to lessen the negative stereotypes about ageing. Another rich area of research related to ageing comes from existential psychology and its ideas about the fundamental existential concern—death (Wong, 2008). Signs of ageing remind individuals of time passing and that they too are not an exception in growing old and eventually dying, which may create anxiety. Indeed, previous studies have found that death anxiety and ageing anxiety were related. Findings from several studies suggest that ageing anxiety and negative ageing perceptions may at least partially be explained by death anxiety given that ageing is a reminder of one's mortality (e.g., Bodner et al., 2015; Kahraman & Erkent, 2023). Other research suggests that some personality traits, such as neuroticism, may be related to more anxiety about ageing (Bryant et al., 2016).

Described quantitative studies usually examine some selected factors as related to ageing attitudes; however, qualitative studies can allow for an exploration of participants' own perceptions about the processes surrounding their ageing acceptance. Some previous qualitative studies have explored certain aspects of ageing perceptions, especially in older adults, for example, successful ageing (Teater & Chonody, 2020) or subjective age (Chonody et al., 2023). These studies found that older adults' views on health, social relationships, meaning, and satisfaction with life, and other aspects are important to their perception of and adjustment to ageing and successful ageing (Chonody et al., 2023; Teater & Chonody, 2020). Other qualitative studies have specifically explored the importance of mobility, health, and physical activity in the context of the ageing experience (Fougner et al., 2019; Goins et al., 2015; Tkatch et al., 2017). Other qualitative studies have sought to understand women's body image, which encompasses one's body-related self-perceptions and self-attitudes (Cash, 2004), in the context of ageing, their anti-ageing practices or motivations for these practices in older adults, and older women specifically (Chung, 2020; Clarke & Griffin, 2007; Jankowski et al., 2016), as well as among young and middle-aged women (Muise & Desmarais, 2010; Wu et al., 2022). Some qualitative studies exploring women's experience and adjustment to menopause have also touched upon the factors related to acceptance of ageing in the mid-life period (Ilankoon et al., 2021; Refaei et al., 2022). These previous studies investigated women's perceptions of specific aspects of their ageing and acceptance of it, but the focus is often just one element of the ageing process rather than a broad focus on what forms their attitudes toward ageing and older adulthood.

While there are many quantitative studies examining ageing perceptions and qualitative studies exploring different aspects of the ageing experience, not many of these studies explored the question of what women in middle adulthood perceive as important factors related to their attitudes toward the ageing process. In particular, studies are lacking in Lithuania, where negative perceptions of ageing and older adults seem to be quite prominent (Rapolienė, 2015; Rychtaříková, 2019). Inside the relatively positive or negative attitudes towards one's ageing might be a more nuanced, continuous, and dynamic process, rather than a single, final position of low or high acceptance. The qualitative method allows us to explore what helps or hinders women's attitudes towards their ageing within different levels of relative acceptance. Given that ageing may mean different things in different life stages, greater insight into the perspective of specifically women in middle adulthood could provide a deeper understanding of important processes in this life stage. Thus, the goal of this study was to explore how women in middle adulthood experience and accept their ageing and what they would name as something helping or hindering ageing acceptance. A qualitative approach highlights women's experiences with ageing; while interpreted by the researcher, it is guided by women's own accounts and emphasis on what factors help or challenge their ageing acceptance.

## Method

This study is part of the wider research project about women's ageing experience in middle adulthood and their attitudes towards ageing. For this paper, our focus is on the perspectives of women on their ageing acceptance and the elements they identify and relate to it. Semi-structured interviews were used to elicit their experiences and perspectives. This also enabled the participants to identify and provide context for their experiences with ageing. The data were analyzed using reflexive thematic analysis (Braun & Clarke, 2006; Braun & Clarke, 2019).

### Participants

The sample included 26 women between the ages of 30 and 57, who were recruited using convenience and snowball sampling by sharing the study invitation on social media pages and groups and snowballing sampling from participants' and researchers' personal and professional contacts. The recruitment announcement detailed individual interviews about one's experiences of ageing and invited women aged 30–59 years to participate. We included women in the age range from the end of young adulthood (Perovic, 2016) to the start of older age (UNHCR, 2025). This life stage can broadly be called middle adulthood, even though the exact age ranges used to define this period vary and are not clearly established (Beyer & Lazzara, 2025; Lachman et al., 2015). We chose a broader middle adulthood span for our study purposes to include a wide range of experiences from different stages of middle adulthood. Most often, participants interested in participating in the study reached out to contact the researcher. However, to reach more participants from rural environments, several women were explicitly invited to participate in the study through the researcher's or previous participants' contacts. As snowballing invitations to participate in the study were done informally through previous participants or other contacts, the refusals to participate were not formally reported and tracked. No participants dropped out after the interviews were arranged or during the interviews. Braun and Clarke (2021) suggested that the arguments for data saturation are not consistent with the reflexive thematic analysis approach and instead suggest approximating the needed sample size to cover the research question in enough depth and reach the desired diversity and then adjusting according to the data richness. We estimated around 20 participants were needed to obtain enough rich data from participants from different sociodemographic groups for our study questions. Recruitment continued because some interviews were shorter, and the inclusion of additional participants without university education and not from an urban environment was desired. During recruitment, an active effort to have a similar number of participants from different age groups was made, as well as the inclusion of diversity in education, geographic location, ethnicity, relationship, and health status. Yet, most participants were highly educated, living in large cities with spouses or partners, and rated their finances and health high. Most of the sample characteristics are found in Table I. Twenty-one participants were working, 2 were not working at the time, 2 were on maternity leave, and 1 was on sick leave. One participant had a disability status. The participants' children's ages ranged from 6 months to 34 years.

**Table I.** Characteristics of the participants (*N* = 26).

Age (years)	
Mean (SD)	44.5 (8.32)
Range	30–57
Age groups, *n*	
30–34	2
35–39	7
40–44	3
45–49	5
50–54	4
55–57	5
Education level, *n*	
University education	18
Higher non university education/secondary education	3
Vocational education, highschool or lower education	5
Residency type, *n*	
Big city	16
Small town	2
Splitting time between big city and small town	2
Village (rural)	6
Financial situation	
Very good	3
Good	15
Average	8
Bad or very bad	0
Marital status, *n*	
Married or cohabitating with a partner	20
Partnering without cohabitation	1
Single	2
Divorced	3
Has children, *n*	
Yes	20
No	6
Has chronic illnesses, *n*	
Yes, significantly affecting life	1
Yes, mildly affecting everyday life	8
No	17
Subjective health rating	
Very good	3
Good	19
Average	4
Bad/very bad	0
Ethnicity	
Lithuanian	22
Russian	2
Other	2

### Data collection and procedure

The study was reviewed and approved by the Lithuanian Centre for Social Sciences Committee on Research Ethics (No. AMTEK-P-2), and data collection as well as other parts of the study were conducted according to the principles of the Helsinki Declaration. Semi-structured interviews with participants were conducted from April to June of 2024 by the first author in neutral spaces to allow for privacy, for example in the Lithuanian Centre for Social Sciences office, university classrooms, and participants' workplace or home. The interview guide was prepared by members of the research team and included around 15 questions about different aspects of participants' experiences and opinions about ageing. The participants were asked to share their thoughts about ageing that come up in their everyday life, and specific questions about why they think they feel some negative emotions about ageing and what helps or would help them to accept ageing were also included (e.g., “Why do you think you feel negative emotions about ageing?”; “What do you think could help you to accept, to come to terms with your ageing?” or “What do you think helps you to have a positive attitude towards your ageing?”). The semi-structured interview allowed the researcher to adapt questions and shape the direction of the interview in response to the participant's answers; however, all key questions were covered in each interview. The length of the interviews varied from 15 to 120 min with an average of 54 minutes. Notably, the shorter interviews (4 interviews were between 15 and 20 min) were with participants who were explicitly asked to participate in the study in contrast to participants who reached out themselves. In these cases, participants may not have elaborated on the answers, answered that they did not think about ageing a lot, or were not concerned about ageing, and some follow-up questions were not relevant in these cases. While collecting the data, it was noted that these interviewers were shorter but were important, as they added to the diversity of the perspectives and experiences of ageing, and we recruited more people from the same age category to compensate for the shorter interviews. Before the interview, the participants were given information about the nature of the study, voluntary participation, data storage, usage and presentation of the results, etc., and participants gave their written consent to participate in the study. After the interview, they completed the demographic information form. Participation in the study was voluntary, and participants were not compensated.

### Theoretical orientation

The goal of our study was to examine the perspectives of the participants. Therefore, we adopted an experiential orientation within reflexive thematic analysis (Braun & Clarke, 2006; Braun & Clarke, 2021). Our analysis focused on the ontological and epistemological position of critical realism (an objective reality can only be partially understood through the subjective perspectives of participants and the researcher's interpretation) and contextualism assumptions (participants' reality is dependent on their social and cultural setting, and so is the researcher's understanding of it) (Braun & Clarke, 2013). Given our experiential approach and the goal of the study to understand participants' perspectives and experiences through their accounts and language, we deliberately did not choose a priori theoretical framework for our analysis. Rather, we used inductive analysis, which is coding guided by data, and both semantic and latent coding, meaning coding directly observable and implicit meanings, were utilized (Braun & Clarke, 2022). We acknowledge that completely pure inductive coding is not realistic, given that a researcher already has knowledge of the field, though to avoid this potential influence, self-reflection processes and supervisions were utilized. Using conceptual theory to frame our questions and analysis would be more suitable for a critical orientation and deductive coding but would have been contradictive to our experiential approach goals and inductive approach (Byrne, 2022). Due to our experiential approach, our analysis and interpretation focused more on describing participants' meanings, opinions, and experiences using their own words rather than making theoretical interpretations about the way that participants construct their experiences, which is more suitable for the critical orientation approach (Braun & Clarke, 2012; Byrne, 2022). After the analysis was completed, we contextualized and interpreted the findings with respect to theoretical perspectives, like the stereotype embodiment theory, social clock theory, existential psychology theories, body image theories, and others.

### Data analysis

Audio recordings of the interviews were transcribed by a professional transcriber who signed a confidentiality agreement. Transcriptions were anonymized or pseudo-anonymized by removing or changing details that could identify participants. The data were then analyzed according to the six steps of reflexive thematic analysis (Braun & Clarke, 2006). The analysis process began with the first author's immersion in the data through conducting interviews, noting preliminary ideas, reviewing transcriptions, and relistening to the material. Early engagement with the data helped identify some recurring patterns, such as comparisons between ageing in Lithuania and abroad, the protective role of close social ties, and links between ageing and thoughts about death. The data were then systematically coded in two stages, with initial codes refined and reorganized into broader categories as conceptual connections emerged. For instance, codes reflecting the importance of parental and peer views of ageing were eventually combined into a single category. Related categories were integrated into subthemes and overarching themes that captured shared meanings—for example, ideas around control in ageing coalesced into subthemes expressing tensions between control and acceptance. Themes were reviewed and discussed collaboratively, after which less substantial subthemes were removed or merged, and the final themes were clearly defined and named to reflect their core meanings. The process concluded with the synthesis of findings into a cohesive report, supported by translated data extracts illustrating key themes and subthemes.

This paper focuses on themes related to ageing acceptance, which was conceptualized as a person's neutral, positive, or negative (as low acceptance) thoughts and feelings about ageing and their attitudes towards ageing. Therefore, all the parts of the interview where the participants were talking about what is related to how they or others feel, think about or accept ageing were coded. All interviews and analyses were carried out in the Lithuanian language. The selected quotes were translated to English using machine translation tools such as DeepL and then adjusted for accuracy, tone, and readability by the first author, who is a native Lithuanian speaker and fluent in English. For the translation, we tried to keep a balance of staying close to the original quote in language and tone but making it readable and understandable in English.

### Trustworthiness

The reflexive thematic analysis approach assumes an active interpretation by the researcher, which is inherently subjective and thus incompatible with some of the traditional credibility checks, such as coding reliability measures (Braun & Clarke, 2022; Braun & Clarke, 2024). Instead, the quality and rigor of the analysis were strengthened through reflexivity throughout the interviewing and data analysis process, such as reflexivity diaries, verbatim transcripts of the interviews, thorough open-ended coding of each data item, and a second round of coding to collate codes to ensure deeper and extended analysis of the data. Moreover, reflecting on how personal views or vivid examples could shape themes or subthemes and check back to original data to ensure that they are grounded and supported by the data, as well as discussing themes and subthemes with team members to deepen and expand understanding, are integral to the data analysis process (Braun & Clarke, 2022; Braun & Clarke, 2024). The first author, who conducted the interviews and performed the coding, is a Lithuanian woman in her mid-thirties, and the other team members are also women who would fit the study's range of middle adulthood, and their own experiences and assumptions towards ageing needed to be reflected during the analysis.

## Results

The analysis resulted in the generation of four main themes and 12 subthemes (see Table II). The main themes were: examples of older figures in the environment; personal attitudes and traits; (no) pressure in the environment; and tension over control of ageing. Each theme and subtheme are explained below and are illustrated by quotes from the participants. The participants' quotes have the participant's code—a letter according to the interview number and their age group. To illustrate how often some ideas were expressed, we used terms like “most of the participants” when loosely more than two-thirds of the participants discussed some ideas, “many” when around half of the participants discussed the idea, and “some” when around one-third or fewer participants expressed certain ideas.

**Table II.** Main themes and subthemes of influences related to ageing acceptance.

Themes	Subthemes
Examples of older figures in the environment	Older family members—the image of an old age
	Negative perception of old age in Lithuania
Personal attitudes and traits	Acceptance of ageing and death as natural and unavoidable
	Satisfaction with one's life
	Positive mindset
	Relationship with oneself and one's appearance
(No) pressure in the environment	Society's negativity about ageing creates pressure
	The personal environment helps to embrace ageing or creates pressure
Tension over control of ageing	You can't stop ageing
	People can and should control their ageing
	Tension of should
	Tension between the pressures of anti-ageing culture and the internal rejection of it

### Examples of older figures in the environment

Almost all participants gave examples of older people in their environment to explain their views on ageing and older age with two sub-themes: older family member—the image of an old age—and negative perception of old age in Lithuania.

*Older family members—the image of an old age.* Most of the participants reflected that their image of ageing and old age was based on seeing up close what family members experienced. Strong positive or negative examples in the environment were identified as important sources of forming either positive or negative views of older individuals, which in turn influenced their attitudes towards ageing. For example, one participant stated:

“*I can't say that I was ever afraid of old age at all. I have had, well, fantastic examples of older people around me with such wisdom of life, with such a calm presence and awareness of the world that it is, well, very, very natural to me”* (R, 55–59).

A few participants said that because their mothers or parents died early, they did not see their ageing process and therefore struggled with their view of ageing. In some cases, women mentioned that they have also seen different or opposite examples of old age in their environment than what they named as the ones that formed their views.

*Negative perceptions of old age in Lithuania.* More than a third of the participants mentioned in one way or another that old age in Lithuania seemed like a difficult experience, especially because older people struggle with finances and isolation. The view that this is specific to Lithuania is illustrated through their comparison of what they see when they travel abroad, which implies Western or Southern Europe. For example, one participant stated:

*<…>whenever my husband and I travel, we see abroad, really, really old people, already with walking sticks, holding hands on a beach somewhere, and we say: “If only we could live like this at an old age...” And I think: “Damn, but will we be able to? Because for us old age is somehow quite a different thing.”* (H, 35–39).

Another participant stated that insecurity of social and medical care at the state level affected how she can accept ageing and that if she lived in a wealthier country where *“there were safeguards that were not only up to me to take care of it, I would be able to grow old and accept it more peacefully”* (M, 40–44).

### Personal attitudes and traits

Most of the participants talked about the personal characteristics and orientations that are related to how they or people in general accept ageing, sometimes even emphasizing how it all depends on a person's personal worldview and personality. This theme had four sub-themes of: acceptance of ageing and death as natural and unavoidable; satisfaction with one's life; positive mindset; and relationship with oneself and one's appearance.

*Acceptance of ageing and death as natural and unavoidable.* Many participants talked about accepting ageing and death as a natural and unavoidable process, which seemed to ease their relationship with it. For example, one participant, when asked what helps her accept ageing, stated:

*The mindset. Well, what to do now that you are getting a bit older? Well, it's [sighs] everybody [stresses] getting old. Everybody. We're [smiles] children, we're all growing up, we're all getting older. We're all going to die, well, so what?* (N, 50–54).

Some participants indicated that nature, religion, philosophy, and psychology helped them accept ageing and death as a natural part of life. However, understanding that ageing is natural does not necessarily mean that it is easy to accept on an emotional level. One participant struggling with her ageing acceptance expressed: *“Although, as I say, I understand that it's, well, like a natural flow. It's going to happen to everyone. [gets teary] It's just that maybe it's hard to admit to yourself”* (Y, 50–54).

*Satisfaction with one's life.* When discussing their attitudes towards ageing or generally what helps people more positively accept ageing, most participants talked about their satisfaction with aspects of their lives that helped them feel less tension over passing time and feel calmer and secure about the future. Many participants expressed how their good relationships with family members were sources of security and support when facing the unknown or how living an active life, such as having an engaging job, hobbies, and activities that they like, leaves no space to think or worry about ageing. Some participants more explicitly talked about the role of finding meaning in their life or feeling that they had already achieved some life goals, which made ageing acceptance easier. For example, one participant stated:

*If any thoughts about it arise, I always remember that saying, that people who fear aging or, in this case, old age, are the ones who did not take something from the previous stage of life that it could have given them. So, I sometimes, kind of, briefly run through a small summary of my own <…>* (U, 55–59).

A few participants talked about the opposite experience in that they discussed how they feel fear for not having started living their life or for not having figured out how they want to live, which added to their complicated feelings around ageing. As one participant described: *“Maybe I'm afraid, you know, of dying like that [get teary] without even realizing what I wanted, or what I was aiming for <…>”* (Y, 50–54).

*Positive mindset.* Some of the participants talked about their general positive mindset towards life, which included an optimistic tendency. This positivity and optimism explained their ability to accept ageing neutrally or positively as just a continuation of this mindset and these traits. One participant explained how her mind has an automatic tendency to look for the bright side in frustrating situations related to ageing:

*<…> you think: “Damn, my legs hurt” you get disappointed. It's just that for me, those frustrations, I process them quickly. Well, I change my perspective quite quickly: “Okay, and what is good?” And I don't even do it consciously. I do it automatically.* (K, 45–49).

*Relationship with oneself and one's appearance.* The participants discussed general patterns of self-acceptance that were important in the relationship with ageing. Several participants stated that appearance was never very important to them or their identity, which made it easier to accept ageing-related changes in appearance and, in turn, ageing itself. A couple of participants talked about how having strong insecurities about their appearance exacerbated as they started to age. For example, one participant stated, *“Maybe it comes from the fact that I've always had this sort of appearance complex, I guess <…> and then, you know, you get some wrinkles, and then maybe you're even less pretty in the eyes of others.”* (D, 35–39). Only one participant explicitly stated that even though she did not give her appearance great importance, she was used to getting attention from others based on her looks, and as she gets older, she needs to adjust to the changes. Several participants also discussed a general ability for self-acceptance as one of the reasons people easily adjust to ageing-related changes: “*I don't know, maybe it is the basic question of accepting oneself? Just how you accept yourself.”* (Z, 45–49). Some participants also stated that it is easier for them to accept the ageing process because they have achieved a better understanding of themselves and their issues and are now more equipped to accept themselves.

### (No) pressure in the environment

Many of the participants talked about their environment's role in adding or easing the tension over ageing, mainly emphasizing pressures around the ageing appearance. This was further expressed through two subthemes: society's negativity about ageing creates pressure, and the personal environment helps to embrace ageing or creates pressure.

*Society's negativity about ageing creates tensions.* Many of the participants talked about the general negativity around ageing in society, and some specifically mentioned the “cult of youth”, which creates tensions with one's ageing. They specifically expressed how social media transmits this pressure through the idealization of a youthful look and its obsession with anti-ageing products and tools. For example, one participant stated:

*I caught myself thinking that even though if I thought that, well, it's beautiful for me when face gradually gets some wisdom, let's say, some wrinkles, but then a need came “Maybe I should mask it? Because what does it look like? Because the whole world is into anti-aging <…>”* (F, 35–39).

Some of the participants also talked about feeling tension over what other people might be thinking when they see signs of their ageing, which is reflective of social norms that negatively judge signs of ageing.

*A personal environment helps to embrace ageing or creates pressure.* A close personal social circle that does not focus too much on appearance or ageing concerns was identified by some of the participants as helping them in accepting ageing, for example,

*So I grew up in an environment where, well, that appearance is not very much emphasized. <…> Girlfriends... the vast majority of them are also, well, like, “Well, you're getting older. It is what it is” and there's no advocacy of some of that current technology.* (D, 35–39).

On the other hand, a few participants expressed how some people in their lives have a complicated or negative views and reactions to ageing, and this added to their own ageing tensions. Some participants also described how one's job might create or relieve pressure about ageing. Most emphasized that an ageing appearance is not a problem at their job; however, one participant expressed that her job puts pressure on her about her appearance: *“I keep asking myself that if I were in a different profession, would I pay attention to it? Would I not put more focus on professional development rather than on how a person sees me at first contact?”* (F, 35–39).

### Tension over control of ageing

Most of the participants breached the idea of control when talking about ageing, often reflecting tensions over expectations for how much control one can have in ageing. These tensions seemed to be related to the ageing acceptance processes. This theme comprises the following four subthemes: You cannot stop ageing; People can and should control their ageing; The tension of should; and Tension between the pressures of anti-ageing culture and the internal rejection of it.

*You cannot stop ageing*. Many of the participants talked about the fact that at the end of the day, it is impossible to stop ageing. One participant explained:

*It's like you want to slow it down, but you understand that you can't stop it. It's like fighting windmills. And it's not clear if it's really worth investing that much or if it would just be easier to accept it as a process <…>* (I, 35–39).

This understanding that ageing cannot be stopped was sometimes mentioned as an uneasy thought, but it also often implied some relief from the notion that something needs to be done about it. A few participants mentioned things such as genetics or external events that are beyond one's control: *“And I think part of those processes are still... also very much genetically determined, and that's just the way it is.”* (C, 35–39).

*People can and should control their ageing*. Many of the participants also talked about the fact that people need to put in effort and energy to have a better ageing experience and they are responsible for feeling better or worse at an older age. For example, one participant stated: *“<…> how you take care of yourself today, that's how you'll look in 10 years and how you'll feel.”* (O, 45–49). Some participants mentioned that when talking about older relatives or other older figures, they thought that something could have been done differently to have better health now. They also explained that feeling that some things can be undone and fixed or that at least they are doing something for their ageing makes them feel better, which also calms them *“Like, for example, doing some facial self-massage or getting a procedure <…> It's like I feel psychologically better that ‘At least I'm doing something.’”* (I, 35–39).

*The tension of should.* Many participants expressed that they know that they should be doing things for better ageing, such as exercising more, eating healthier, and saving money. Often, they were trying to do so, but a sentiment of not doing it enough or that it could be done more was often expressed. As one participant put it: *“<…> basically it still comes down to lifestyle [smiles], and it's not always that easy to stick to all those principles of a healthy lifestyle, even though you know them perfectly [smiles]”* (J, 30–34). Even though participants knew what should be done, actually making themselves do it was the hard part. For example, one participant stated: *“I advise my mother, for example, but maybe I don't even do it myself. It's very easy to give advice [laughs]”* (E, 55–59).

*The inner tension between the anti-ageing culture's pressures and the rejection of it.* Some participants explicitly stated or implied the inner tension between the two. On the one hand, feeling the pressure of society's cult of youth and the need to do something to “stop” or slow down signs of ageing. On the other hand, they want to reject this anti-ageing attitude and embrace and accept their ageing. One participant stated:

*There's a little bit of an internal tension because it's kind of hard to find that line where I still want to declare that it's natural and it's okay and I don't want to do anything about it, and where it's a kind of a social expectation and a norm that you want to meet.* (C, 35–39).

Another participant discussed how one has to find their personal line on the spectrum between complete acceptance and intense appearance control, which also reflects this inner tension. A few participants expressed that the availability of anti-ageing procedures and normalization of their use created tension, as it made them wonder if they should do something about their ageing appearance.

## Discussion

This study aimed to explore what helps or hinders ageing acceptance according to women in middle adulthood. The analysis generated four main themes that women mention when discussing influences on their acceptance of ageing: examples of older figures in the environment, personal attitudes and traits, (no) pressure in the environment, and tension over control of ageing. Each theme is presented in relation to its significance to the existing body of knowledge on this topic and implications for addressing the ageing acceptance process among women.

When discussing ageing and old age, many participants either implied or explicitly stated that the type of examples they saw in their close environment formed either positive or negative views of older age and, in turn, seemed to be related to participants' attitudes towards ageing and moving towards older age. In the ageing anxiety literature, the contact, and quality of the contact often seem to be described as having a somewhat secondary role in combating or reinforcing an already-formed image of old age based on stereotypes prevalent in society (Barnett & Adams, 2018; Bousfield & Hutchison, 2010). In our study, close older figures were emphasized as major sources of old-age image, suggesting that the role of close examples might be greater than what is usually discussed in the literature, such as ageing stereotype embodiment theory, where society's views on ageing are emphasized as creating old-age stereotypes (Levy, 2009). Several participants describing not having a strong image of old age and ageing, because they did not have examples in a close environment, would seem to support this idea. However, as women sometimes passingly mentioned that they also had opposite or diverse examples in their surroundings, it might be that it is not a straightforward process of how examples influence their image of old age. There could be a negativity bias—more attention might be given to negative examples of older age than neutral or positive ones. It also might be that participants' personality traits, attitudes, or already formed views influenced which examples they noticed and paid more attention to.

It seems that economic, social issues, and other issues in the Lithuanian older population were related to how women were seeing older adults, older age, and their feelings about ageing. Several reports show a large gap between younger and older Lithuanian cohorts in happiness levels (OECD, 2022), social exclusion, and poverty levels (Eurostat, 2019), which reveals a significant gap between younger and older adults in several quality-of-life measures. Our study results seem to show that women in Lithuania were perceiving this gap and difficulties of the older generation and they were creating concerns about their own future in this age group. Compared to other European countries, a large proportion of older adults in Lithuania see age as limiting their ability to do what they want, and Lithuania had the highest percentage of older adults naming financial reasons for not participating in cultural and sports activities among all European Union countries (OECD, 2022)—a connection made by at least few participants when discussing older age in Lithuania. Previous studies also found elevated negative views of older adults and ageism in Lithuania (Rapolienė, 2015; Rychtaříková, 2019). Our findings illustrate one of the possible, yet less widely discussed and emphasized, pathways between economic and social difficulties in the country's older adult population, affecting the perception of older age and, in turn, personal ageing anxiety. Future cross-cultural studies could examine these pathways to further elucidate their role in explaining the acceptance of ageing.

The theme of personal attitudes and traits reflects underlying personal characteristics that seem to influence how women perceive and accept ageing. For several participants, their view of ageing or death as something natural and unavoidable was something that helped them to have more peaceful views and emotions towards ageing, but this acceptance of death and ageing as natural and inevitable is something deeper than their knowledge of the fact but also their relative emotional ease about it (Wong et al., 1994). The fact that some participants talked about ageing acceptance, closely connecting it to death acceptance, is not surprising, as ageing anxiety is closely related to death anxiety in the literature and research (Bodner et al., 2015; Kahraman & Erkent, 2023). Women also named satisfaction in or about their lives as something that eases their ageing anxiety. Quality of life has also been found to be related to perceptions of one's own ageing among older adults (Velaithan et al., 2024), though often opposite directionality is implied in the interpretations of the studies. Women talking about living an active and meaningful life as easing their views to ageing can also be understood in the context of existential psychology theories, as some authors have suggested that fear of death or life passing is related to how guilty they might feel about not using their potential and living to the fullest (Wong, 2008). This finding might also be interpreted within the social clock theory (Neugarten, 1979), which suggests that people are aware of society's prescribed timeline for major life events, are monitoring their own progress, and their perceived failures to meet these timelines and goals can induce anxiety around ageing (Momtaz et al., 2021). Our findings suggest that participants' perceived success at meeting some of these goals might be related to their perceived ease of tension around ageing. Our findings suggest that it might be worth studying the relationship between quality of life or satisfaction with life and perceptions of ageing in a more nuanced way. For example, exploring the directionality of the relationship between the two and the specific modifiable aspects to increase life satisfaction and, in turn, positive ageing perception.

The participants mentioned that they responded to ageing with the same patterns that they respond to different things in life is not surprising, as personality traits are defined as relatively enduring patterns of thoughts, feelings, and actions across different life domains (McCrae & Costa Jr., 1999). Neuroticism has been most consistently found to be related to ageing attitudes, but some evidence also suggests that higher extraversion, optimism, and, in some instances, consciousness are related to lower ageing anxiety and more positive ageing attitudes (Barnett & Adams, 2018; Bryant et al., 2016; Wettstein et al., 2020). What is more, personality traits such as lower neuroticism, higher optimism, and generally a more positive mindset might also be underlying and related to other subthemes—easier emotional acceptance of death and ageing as inevitable, subjective satisfaction with life, and more self-acceptance.

Women in the study talked about the relationship with one's appearance and one's relationship with oneself as something related to how a person would accept ageing. The participants discussed that appearance not being very important to them helped them accept ageing more easily, while participants who reported a history of a negative relationship with their appearance reported that the ageing process was harder because of it. In the body image research field and theories, appearance importance is often, but not always, related to appearance dissatisfaction (Jarry et al., 2019). In line with our results, two previous studies have found that appearance investment and importance are related to physical appearance ageing anxiety (Carrard et al., 2021; Slevec & Tiggemann, 2010). The participants also discussed the wider ability of self-acceptance, and a closely related construct of self-compassion has been found to be related to lower ageing anxiety among older adults in one previous study (Delkhah et al., 2023) but is scarcely studied in ageing anxiety research. Self-compassion was also found to be consistently related to a more positive relationship with one's body (Braun et al., 2016). Similarly, a review of qualitative studies found that older adults would talk about the importance of the ability to accept one's current situation, including limitations associated with ageing for successful ageing (Teater & Chonody, 2020). Higher self-acceptance and related self-compassion might mean that women can accept changes and some losses that can accompany ageing with more ease and self-kindness. The results of our study highlight that questions related to body image and self-acceptance are relevant to the ageing acceptance process during middle adulthood and may be important for further research in this context, expanding the focus beyond the more typical context of body size and shape.

Unsurprisingly, participants talked about seeing and feeling pressure in society from the way ageing is talked about and portrayed. The heavy weight of society's cult of youth and wide negative views of ageing and older age on people's experiences of ageing is a widely talked about and recognized issue (Levy, 2009). The participants also discussed that views on appearance and ageing in the social bubble of a person were also creating pressure and tension or acceptance of their ageing. It could be seen as something “socially contagious”—if a person's immediate social surrounding is feeling ageing anxiety, is concerned about ageing signs, and working on fixing them, then these anxieties and concerns might also start affecting the person. In a previous qualitative study exploring body image and acceptance of cosmetic surgery, participants also discussed appearance-focused peer groups as a source of pressure for each other and encouragement of cosmetic procedures (Wu et al., 2022). Another study found that participants' appearance ageing anxiety levels were related to their perceived pressure from media and other people to maintain or improve their appearance (Muise & Desmarais, 2010). However, our study results seem to suggest that a close personal environment not being overly concerned about ageing might relieve some pressures created by wider anti-ageing pressures for some people. This might be another important direction to consider in attitudes toward ageing research, as the influence of a close social circle's perception of ageing seems to be rarely studied.

The results of our study seem to reveal the complicated nature of control in the process of ageing. It is possible that the nature of ageing where both are true—one cannot stop ageing but can also affect the ageing process, creates tension of the level of control one should try to take in the ageing process. If a person tries too much to control what is unavoidable, it is like “fighting windmills” and might create constant pressure, anxiety, and disappointment. However, a person might feel that if they do not put effort into doing what they can, they might have a worse ageing experience. As discussed previously, older adults expressed the ability to accept and adapt to the current ageing situation as part of successful ageing (Teater & Chonody, 2020), on the other hand, studies have shown that some actions are dependent on the person, for example, healthy living habits are related to successful ageing in older individuals (Lin et al., 2020). This theme, in a way, touches upon some ideas of theories describing adaptation to ageing like the selective optimization with compensation model (Baltes & Carstensen,^1996^) and a two-process framework (Brandtstädter & Rothermund, 2002), which explain how coping with ageing involves two processes: trying to modify the situation so it matches personal goals and adjusting to the situation when goals become unreachable.

Our findings highlight the tension of finding the right balance in these processes and the tension of external pressure to set specific goals, such as remaining youthful, when they do not match internal values. A few previous studies of women's anti-ageing procedure use (Chung, 2020; Clarke & Griffin, 2007; Muise & Desmarais, 2010) also found that women talking about the tension between acceptance of ageing and resisting it, they also named tension over using anti-ageing procedures and acceptance of natural ageing appearance, as well as trying to define what is natural ageing for them. In the Jankowski et al. (2016) study of older adults, the authors reported similar tensions in managing different pressures regarding ageing, but they also reported feeling pressure to age gracefully, naturally, and appropriately to their age. Traditionally, a sense of control is found to be associated with more positive life outcomes (Cheng et al., 2016) as well as with quality of life and well-being in older adults more specifically (Ingrand et al., 2018). However, further examining specific beliefs related to control and the appearance of ageing in middle adulthood, given that previous research shows a complicated picture, and appearance control beliefs can be related to better well-being outcomes but can also backfire, for example, resulting in internalized weight stigma and contradictory eating behaviours (Boursier et al., 2020). The results of our study suggested that a sense of control in relationship to one's ageing might be complex and subtle, where a balance between self-acceptance, acceptance, and a sense of control over one's ageing is intertwined and not so straightforward.

Our study analyzed important themes from participants across a wide age range in middle adulthood, and notably, all themes and most subthemes were discussed by women in their 30s, 40s, and 50s, indicating that these processes related to ageing acceptance seem to be relevant throughout this life period. However, we did note a difference in the prominence of some subthemes across different age groups. For example, the subthemes of pressure of society's negative views of ageing and internal tension between the pressure of anti-ageing culture and internal rejection were not discussed as much by women in their 50s. This might represent what is sometimes hypothesized in the literature, that younger people are more affected by ageing stereotypes and hold more negative ageing attitudes, while the effect of these stereotypes might be mitigated as more real-life experience of ageing is gained (Donizzetti, 2019). It might be that more experience of ageing and going through the second half of the middle adulthood might also give time and occasions to accept ageing and death as something natural and unavoidable on a deeper level, as the subtheme around this acceptance was more prominently discussed by participants in their 40s and 50s. However, since our analysis was not aimed at exploring participants' experiences in different age groups, a more extensive and deeper analysis should be performed in a separate study or analysis of the data.

### Limitations

The results of our study need to be interpreted while keeping in mind study limitations. One of the study's sample's limitations might have been a self-selection bias, as more motivated, generally active, and stronger prosocial tendencies might have led to some women being more interested in participating in the study. This could have been reflected in the results, as some themes have more aspects about positive acceptance of ageing than trouble accepting ageing. Women with more negative attitudes towards ageing might have been less prone to participate in the study. The study included the recruitment of participants from diverse socioeconomic backgrounds, yet most women were highly educated and from large cities. What is more, a bigger part of the participants from rural settings were invited explicitly to participate in the study instead of contacting the research team themselves. These interviews were more often shorter, as participants might have had less interest or motivation in the topic or the study, so data from this group of participants might not have been as prominent in the analysis. Another important context of the study was the inclusion of a wide age range to constitute the middle adulthood period. Women in different decades of middle adulthood have different experiences and perspectives, and it is important to capture the patterns in the whole range of the period between young adulthood and older age. Future studies could explore different themes and subthemes emerging from different stages of women's middle adulthood. Future research could also explore different dimensions of these questions using different qualitative approaches, such as grounded theory or phenomenology. Our study was performed in the Lithuanian context, and Lithuania-specific content emerged as one of the subthemes. While it is important to have that in mind when interpreting results and ageing perception is thought to be closely related to cultural influences, some previous studies have shown that ageing stereotypes do not differ significantly among different cultures (De Paula Couto et al., 2022), and a recent study in Lithuania found structure of the ageing anxiety measure shows similar patterns to what is found in other countries (Gegieckaitė et al., 2025), suggesting that ageing anxiety in Lithuania might be experienced in a similar way to other cultures.

## Conclusions

This study illustrated the perspectives of women in middle adulthood on what is related to their attitudes toward ageing and their ageing acceptance. Four main themes for ageing acceptance were found, which included the importance of older adults' examples, personal traits and attitudes, pressure in the environment, and ageing control questions. A better understanding of the processes that help or hinder women's perceptions and emotional reactions to ageing in middle adulthood means that interventions can be developed to create a better present and future ageing experience. For example, interventions and community programs that foster intergenerational contact could counteract negative views and promote more positive and diverse images of older adults. Public health campaigns and educational initiatives could focus on integrating messages that normalize ageing and reframe it as a natural and meaningful life stage rather than a decline to be avoided. Incorporating self-compassion and body acceptance interventions into health promotion and psychological support services could help women develop more adaptive views and emotional reactions to ageing and body changes. Moreover, some issues, such as tension or the balance between self-acceptance and control in ageing, should be studied further to understand their more nuanced effects in relation to one's own ageing. Finally, addressing the structural insecurities that heighten fears of ageing, such as financial instability and inadequate health and social care resources and services, could reduce anxiety and promote greater acceptance of ageing. Cultivating self-acceptance, fostering positive models of older adults, and reducing environmental pressures on ageing can support women's mental health, social participation, and healthy ageing at the individual, communal, and societal levels.

## Data Availability

The extended theme and subtheme scheme with supporting quotes is available from the corresponding author, G.G., upon reasonable request. Full interview transcripts will not be publicly available, as they could compromise the privacy of research participants, and their given informed consent did not include permission to share full transcripts of the interviews.

## References

[cit0001] Baltes, M. M., & Carstensen, L. L. (1996). The process of successful ageing. *Ageing and Society*, *16*(4), 397–422. 10.1017/S0144686X00003603

[cit0002] Barrett, A. E., & Toothman, E. L. (2018). Multiple “old ages”: The influence of social context on women's aging anxiety. *The Journals of Gerontology: Series B*, *73*(8), e154–e164. 10.1093/geronb/gbx027 PMC6178960 28453655

[cit0003] Barnett, M. D., & Adams, C. M. (2018). Ageism and aging anxiety among young adults: Relationships with contact, knowledge, fear of death, and optimism. *Educational Gerontology*, *44*(11), 693–700. 10.1080/03601277.2018.1537163

[cit0004] Beyer, A., & Lazzara, J. (2025). *Psychology through the lifespan*. Open Maricopa. Retrieved from https://open.maricopa.edu/psy240mm/

[cit0005] Bousfield, C., & Hutchison, P. (2010). Contact, anxiety, and young people's attitudes and behavioral intentions towards the elderly. *Educational Gerontology*, *36*(6), 451–466. 10.1080/03601270903324362

[cit0006] Bodner, E., Shrira, A., Bergman, Y. S., Cohen-Fridel, S., & Grossman, E. S. (2015). The interaction between aging and death anxieties predicts ageism. *Personality and Individual Differences*, *86*, 15–19. 10.1016/j.paid.2015.05.022

[cit0007] Boursier, V., Gioia, F., & Griffiths, M. D. (2020). Objectified body consciousness, body image control in photos, and problematic social networking: The role of appearance control beliefs. *Frontiers in Psychology*, *11*, 147. 10.3389/fpsyg.2020.00147 32158409 PMC7052303

[cit0008] Brandtstädter, J., & Rothermund, K. (2002). The life-course dynamics of goal pursuit and goal adjustment: A two-process framework. *Developmental Review*, *22*(1), 117–150. 10.1006/drev.2001.0539

[cit0009] Braun, V., & Clarke, V. (2006). Using thematic analysis in psychology. *Qualitative Research in Psychology*, *3*(2), 77–101. 10.1191/1478088706qp063oa

[cit0010] Braun, V., & Clarke, V. (2012). Thematic analysis. In H. Cooper, P. M. Camic, D. L. Long, A. T. Panter, D. Rindskopf, & K. J. Sher (Eds.), *APA handbook of research methods in psychology, Research designs: Quantitative, qualitative, neuropsychological, and biological* (Vol. *2*, pp. 57–71). American Psychological Association. 10.1037/13620-004

[cit0011] Braun, V., & Clarke, V. (2013). *Successful qualitative research: A practical guide for beginners*. Sage Publications.

[cit0012] Braun, V., & Clarke, V. (2019). Reflecting on reflexive thematic analysis. *Qualitative Research in Sport, Exercise and Health*, *11*(4), 589–597. 10.1080/2159676X.2019.1628806

[cit0013] Braun, V., & Clarke, V. (2021). To saturate or not to saturate? Questioning data saturation as a useful concept for thematic analysis and sample-size rationales. *Qualitative Research in Sport, Exercise and Health*, *13*(2), 201–216. 10.1080/2159676X.2019.1704846

[cit0014] Braun, V., & Clarke, V. (2022). Conceptual and design thinking for thematic analysis. *Qualitative Psychology*, *9*(1), 3–26. 10.1037/qup0000196

[cit0015] Braun, V., & Clarke, V. (2024). Supporting best practice in reflexive thematic analysis reporting in palliative medicine: A review of published research and introduction to the Reflexive Thematic Analysis Reporting Guidelines (RTARG). *Palliative Medicine*, *38*(6), 608–616. 10.1177/02692163241234800 38469804 PMC11157981

[cit0016] Braun, T. D., Park, C. L., & Gorin, A. (2016). Self-compassion, body image, and disordered eating: A review of the literature. *Body image*, *17*, 117–131. 10.1016/j.bodyim.2016.03.003 27038782

[cit0017] Brunton, R. J., & Scott, G. (2015). Do we fear ageing? A multidimensional approach to ageing anxiety. *Educational Gerontology*, *41*(11), 786–799. 10.1080/03601277.2015.1050870

[cit0018] Bryant, C., Bei, B., Gilson, K. M., Komiti, A., Jackson, H., & Judd, F. (2016). Antecedents of attitudes to aging: A study of the roles of personality and well-being. *The Gerontologist*, *56*(2), 256–265. 10.1093/geront/gnu041 24793646

[cit0019] Byrne, D. (2022). A worked example of Braun and Clarke's approach to reflexive thematic analysis. *Quality & Quantity*, *56*(3), 1391–1412. 10.1007/s11135-021-01182-y

[cit0020] Carrard, I., Argyrides, M., Ioannou, X., Kvalem, I. L., Waldherr, K., Harcourt, D., & McArdle, S. (2021). Associations between body dissatisfaction, importance of appearance, and aging anxiety with depression, and appearance-related behaviors in women in mid-life. *Journal of Women & Aging*, *33*(1), 70–83. 10.1080/08952841.2019.1681882 31635544

[cit0021] Cash, T. F. (2004). Body image: Past, present, and future. *Body Image*, *1*(1), 1–5. 10.1016/S1740-1445(03)00011-1 18089136

[cit0022] Cheng, C., Cheung, M. W. L., & Lo, B. C. (2016). Relationship of health locus of control with specific health behaviours and global health appraisal: a meta-analysis and effects of moderators. *Health Psychology Review*, *10*(4), 460–477. 10.1080/17437199.2016.1219672 27556686 PMC5214986

[cit0023] Chonody, J. M., Kotzian, A., Godinez, K., Helm, H., Teater, B., & Wang, D. (2023). “I'm not old, just aging”: Perceptions of subjective age and aging among community-dwelling older adults. *Journal of Aging & Social Change*, *13*(1), 89–105. 10.18848/2576-5310/CGP/v13i01/89-105

[cit0024] Chonody, J. M., & Teater, B. (2016). Why do I dread looking old?: A test of social identity theory, terror management theory, and the double standard of aging. *Journal of Women & Aging*, *28*(2), 112–126. 10.1080/08952841.2014.950533 26716474

[cit0025] Chung, S. (2020). Resistance and acceptance: Ambivalent attitudes toward the aging body and antiaging practices among older Korean migrants living in New Zealand. *Journal of Women & Aging*, *32*(3), 259–278. 10.1080/08952841.2018.1529475 30307802

[cit0026] Clarke, L. H., & Griffin, M. (2007). The body natural and the body unnatural: Beauty work and aging. *Journal of Aging Studies*, *21*(3), 187–201. 10.1016/j.jaging.2006.11.001

[cit0027] Cooney, C., Minahan, J., & Siedlecki, K. L. (2021). Do feelings and knowledge about aging predict ageism?. *Journal of Applied Gerontology*, *40*(1), 28–37. 10.1177/0733464819897526 31920128

[cit0028] De Paula Couto, C., Ostermeier, R., & Rothermund, K. (2022). Age differences in age stereotypes: The role of life domain and cultural context. *GeroPsych*, *35*(4), 177–188. 10.1024/1662-9647/a000272

[cit0029] Delkhah, Z., Alivandi Vafa, M., & Moheb, N. (2023). Fear of aging in older adults: The role of meaning in life, self-compassion and perceived social support. *Aging Psychology*, *9*(3), 311–326. 10.22126/jap.2023.9250.1710

[cit0030] Donizzetti, A. R. (2019). Ageism in an aging society: The role of knowledge, anxiety about aging, and stereotypes in young people and adults. *International Journal of Environmental Research and Public Health*, *16*(8), 1329. 10.3390/ijerph16081329 31013873 PMC6517915

[cit0031] Eurostat. (2019, January). *People at risk of poverty or social exclusion*. Eurostat Statistics Explained. Retrieved from https://ec.europa.eu/Eurostat/statistics-explained/index.php?title=Archive

[cit0032] Fougner, M., Bergland, A., Lund, A., & Debesay, J. (2019). Aging and exercise: Perceptions of the active lived-body. *Physiotherapy Theory and Practice*, *35*(7), 651–662. 10.1080/09593985.2018.1456584 29601232

[cit0033] Gegieckaitė, G., Petraškaitė, K., & Zamalijeva, O. (2025). Psychometric properties of the anxiety about aging scale (AAS) in Lithuanian adults. *International Journal of Aging & Human Development*. 10.1177/00914150241313359 PMC12963468 39819079

[cit0034] Goins, R. T., Jones, J., Schure, M., Rosenberg, D. E., Phelan, E. A., Dodson, S., & Jones, D. L. (2015). Older adults' perceptions of mobility: A metasynthesis of qualitative studies. *The Gerontologist*, *55*(6), 929–942. 10.1093/geront/gnu014 24637252

[cit0035] Hess, T. M. (2006). Attitudes toward aging and their effects on behavior. In J. E. Birren, & K. W. Schaie (Eds.), *Handbook of the psychology of aging* (pp. 379–406, 6th ed.). Academic Press.

[cit0036] Ilankoon, I. M. P. S., Samarasinghe, K., & Elgán, C. (2021). Menopause is a natural stage of aging: a qualitative study. *BMC Women's Health*, *21*(1), 47. 10.1186/s12905-020-01164-6 33522914 PMC7849153

[cit0037] Ingrand, I., Paccalin, M., Liuu, E., Gil, R., & Ingrand, P. (2018). Positive perception of aging is a key predictor of quality-of-life in aging people. *PLoS One*, *13*(10), e0204044. 10.1371/journal.pone.0204044 30281672 PMC6169874

[cit0038] Jankowski, G. S., Diedrichs, P. C., Williamson, H., Christopher, G., & Harcourt, D. (2016). Looking age-appropriate while growing old gracefully: A qualitative study of ageing and body image among older adults. *Journal of Health Psychology*, *21*(4), 550–561. 10.1177/1359105314531468 24776689

[cit0039] Jarry, J. L., Dignard, N. A., & O'Driscoll, L. M. (2019). Appearance investment: The construct that changed the field of body image. *Body Image*, *31*, 221–244. 10.1016/j.bodyim.2019.09.001 31653567

[cit0040] Kahraman, S., & Erkent, D. (2023). The mediator role of attitude towards aging and elderliness in the effect of the meaning and purpose of life on death anxiety. *Current Psychology*, *42*(23), 19518–19525. 10.1007/s12144-022-03087-x PMC9010449 35440863

[cit0041] Korkmaz Aslan, G., Kartal, A., Özen Çınar, İ., & Koştu, N. (2017). The relationship between attitudes toward aging and health‐promoting behaviours in older adults. *International Journal of Nursing Practice*, *23*(6), e12594. 10.1111/ijn.12594/29027314

[cit0042] Kornadt, A. E., Siebert, J. S., & Wahl, H. W. (2019). The interplay of personality and attitudes toward own aging across two decades of later life. *PLoS One*, *14*(10), e0223622. 10.1371/journal.pone.0223622 31596876 PMC6785129

[cit0043] Kornadt, A. E., Voss, P., & Rothermund, K. (2015). Hope for the best, prepare for the worst? Future self-views and preparation for age-related changes. *Psychology and Aging*, *30*(4), 967–976. 10.1037/pag0000058 26302028

[cit0044] Lachman, M. E., Teshale, S., & Agrigoroaei, S. (2015). Midlife as a pivotal period in the life course: Balancing growth and decline at the crossroads of youth and old age. *International Journal of Behavioral Development*, *39*(1), 20–31. 10.1177/0165025414533223 25580043 PMC4286887

[cit0045] Lasher, K. P., & Faulkender, P. J. (1993). Measurement of aging anxiety: Development of the anxiety about aging scale. *The International Journal of Aging and Human Development*, *37*(4), 247–259. 10.2190/1U69-9AU2-V6LH-9Y1L 8307644

[cit0046] Levy, B. (2009). Stereotype embodiment: A psychosocial approach to aging. *Current Directions in Psychological Science*, *18*(6), 332–336. 10.1111/j.1467-8721.2009.01662.x 20802838 PMC2927354

[cit0047] Lin, Y. H., Chen, Y. C., Tseng, Y. C., Tsai, S. T., & Tseng, Y. H. (2020). Physical activity and successful aging among middle-aged and older adults: A systematic review and meta-analysis of cohort studies. *Aging*, *12*(9), 7704–7716. 10.18632/aging.103057 32350152 PMC7244057

[cit0048] McCrae, R. R., & Costa Jr., P. T. (1999). A five-factor theory of personality. In L. A. Pervin, & O. P. John (Eds.), *Handbook of personality psychology* (pp. 139–153). Guilford.

[cit0049] Momtaz, Y. A., Mahmoudi, N., & Zanjari, N. (2021). Why do people fear of aging? A theoretical framework. *Advances in Gerontology*, *11*(2), 121–125. 10.1134/S2079057021020089

[cit0050] Mrazek, M. D., Dow, B. R., Richelle, J., Pasch, A. M., Godderis, N., Pamensky, T. A., Rutila, B. A., & Mrazek, A. J. (2024). Aspects of acceptance: Building a shared conceptual understanding. *Frontiers in Psychology*, *15*, 1423976. 10.3389/fpsyg.2024.1423976 38974104 PMC11225620

[cit0051] Muise, A., & Desmarais, S. (2010). Women's perceptions and use of “anti-aging” products. *Sex Roles*, *63*(3–4), 126–137. 10.1007/s11199-010-9791-5

[cit0052] Neugarten, B. L. (1979). Time, age, and the life cycle. *The American Journal of Psychiatry*, *136*(7), 887–894. 10.1176/ajp.136.7.887 453348

[cit0053] OECD. (2022). *A report on the assessment of the current situation of active ageing in Lithuania.* https://www.OECD.org/content/dam/OECD/en/topics/policy-issues/ageing-and-employment/assessment-current-situation-lithuania.pdf

[cit0054] Perovic, B. (2016). Defining youth in contemporary national legal and policy frameworks across Europe. *Partnership between the European Commission and the Council of Europe in the field of youth*. https://pjp-eu.coe.int/en/web/youth-partnership/youth-knowledge-and-policy

[cit0055] Rapolienė, G. (2015). Old age stigmatization. *European quarterly of political attitudes and mentalities*, *4*(1), 63–81. https://nbn-resolving.org/urn:nbn:de:0168-ssoar-416068

[cit0056] Refaei, M., Mardanpour, S., Masoumi, S. Z., & Parsa, P. (2022). Women's experiences in the transition to menopause: a qualitative research. *BMC Women's Health*, *22*(1), 53. 10.1186/s12905-022-01633-0 35219295 PMC8882304

[cit0057] Rychtaříková, J. (2019). Perception of population ageing and age discrimination across EU countries. *Population and Economics*, *3*(4), 1–29. 10.3897/popecon.3.e49760

[cit0058] Slevec, J., & Tiggemann, M. (2010). Attitudes toward cosmetic surgery in middle-aged women: Body image, aging anxiety, and the media. *Psychology of Women Quarterly*, *34*(1), 65–74. 10.1111/j.1471-6402.2009.01542.x

[cit0059] Tajfel, H., & Turner, J. C. (1986). The social identity theory of intergroup behaviour. In S. Worchel, & W. G. Austin (Eds.), *Psychology of intergroup relations* (pp. 7–24). Nelson-Hall.

[cit0060] Teater, B., & Chonody, J. M. (2020). How do older adults define successful aging? A scoping review. *The International Journal of Aging and Human Development*, *91*(4), 599–625. 10.1177/0091415019871207 31456410

[cit0061] Tkatch, R., Musich, S., MacLeod, S., Kraemer, S., Hawkins, K., Wicker, E. R., & Armstrong, D. G. (2017). A qualitative study to examine older adults' perceptions of health: Keys to aging successfully. *Geriatric Nursing*, *38*(6), 485–490. 10.1016/j.gerinurse.2017.02.009 28341064

[cit0062] UNHCR. (2025, June 13). *Older persons* [Webpage]. UNHCR emergency handbook. https://emergency.UNHCR.org/protection/persons-risk/older-persons

[cit0063] Velaithan, V., Tan, M. M., Yu, T. F., Liem, A., Teh, P. L., & Su, T. T. (2024). The association of self-perception of aging and quality of life in older adults: A systematic review. *The Gerontologist*, *64*(4), gnad041. 10.1093/geront/gnad041 37029753 PMC10943510

[cit0064] Wahl, H. W., Drewelies, J., Düzel, S., Lachman, M. E., Smith, J., Eibich, P., Steinhagen-Thiessen, E., Demuth, I., Lindenberger, U., Wagner, G. G., & Ram, N. (2022). Subjective age and attitudes toward own aging across two decades of historical time. *Psychology and Aging*, *37*(3), 413–428. 10.1037/pag0000662 34694838 PMC9487183

[cit0065] Westerhof, G. J., Nehrkorn-Bailey, A. M., Tseng, H. Y., Brothers, A., Siebert, J. S., Wurm, S., Wahl, H. W., & Diehl, M. (2023). Longitudinal effects of subjective aging on health and longevity: An updated meta-analysis. *Psychology and Aging*, *38*, 147–166. 10.1037/pag0000737 36972091 PMC10192139

[cit0066] Wettstein, M., Wahl, H. W., & Siebert, J. S. (2020). 20-year trajectories of health in midlife and old age: Contrasting the impact of personality and attitudes toward own aging. *Psychology and Aging*, *35*(6), 910–924. 10.1037/pag0000464 32271067

[cit0067] Williams, J. C., & Lynn, S. J. (2010). Acceptance: An historical and conceptual review. *Imagination, Cognition and Personality*, *30*(1), 5–56. 10.2190/IC.30.1.c

[cit0068] Wong, P. T. P. (2008). Meaning management theory and death acceptance. In In A Tomer, E. Grafton, & P. T. P. Wong (Eds.), *Existential and Spiritual Issues in Death Attitudes* (pp. 65–87). Erlbaum.

[cit0069] Wong, P. T. P., Reker, G. T., & Gesser, G. (1994). Death Attitude Profile—Revised: A multidimensional measure of attitudes toward death. In R. A. Neimeyer (Ed.), *Death anxiety handbook: Research, instrumentation, and application* (pp. 121–148). Taylor & Francis.

[cit0070] Wu, Y., Mulkens, S., & Alleva, J. M. (2022). Body image and acceptance of cosmetic surgery in China and the Netherlands: A qualitative study on cultural differences and similarities. *Body Image*, *40*, 30–49. 10.1016/j.bodyim.2021.10.007 34801810

